# The validity of ultrasound and shear wave elastography to assess the quality of the rotator cuff

**DOI:** 10.1007/s00330-023-10037-z

**Published:** 2023-08-30

**Authors:** Nina H. C. Peeters, Annick M. van der Kraats, Thomas E. van der Krieken, Dave van Iersel, Esther R. C. Janssen, Frederik O. Lambers Heerspink

**Affiliations:** 1 Department of Radiology, VieCuri Medical Center, Venlo, The Netherlands; 2 Department of Orthopaedic Surgery, VieCuri Medical Center, Tegelseweg 210, 5912 BL Venlo, The Netherlands; 3 Department of Orthopaedic Surgery, Maastricht University Medical Center+, Maastricht, The Netherlands

**Keywords:** Fatty infiltration, Muscle atrophy, Elastography, Ultrasound imaging, Rotator cuff

## Abstract

**Objectives:**

US with shear wave elastography (SWE) could reduce the burden and costs of the diagnostic process for patients with rotator cuff disorders. The aim of this study is to investigate the validity of US and SWE in preoperative assessment of fatty infiltration (FI) and muscle atrophy of the supraspinatus (SSP) and infraspinatus (ISP) muscles.

**Methods:**

Patients with a rotator cuff disorder and a recent shoulder CT or MRI scan were eligible to participate. Goutallier and Warner stages of the SSP and ISP muscle were measured on the scan, for assessment of FI and muscle atrophy, respectively. These findings were compared with shear wave velocities (SWVs) assessed on US. Visual assessment of FI on US was compared with the Goutallier stage. To quantify the amount of muscle atrophy, the occupation ratio between SSP fossa and muscle was measured on MRI and US.

**Results:**

Seventy-eight shoulders were included in the analysis. The correlation found between the occupation ratio on US and Warner and Goutallier stage and ratio on MRI ranged between *r* =  − 0.550 to 0.589. The Goutallier stage of ISP and SSP muscle assessed on US showed a fair correlation with the Goutallier stage on a scan of *r* = 0.574 and *r* = 0.582, respectively. There was a poor correlation between the SWVs and scan results (*r* =  − 0.116 to 0.07*).*

**Conclusion:**

SWE is not a valid method to measure the amount of FI or muscle atrophy in the SSP muscle. Therefore, SWE is not a suitable alternative for MRI in standard preoperative diagnostics in rotator cuff pathologies.

**Clinical relevance statement:**

Shear wave elastography should not be used in the diagnostics of rotator cuff pathologies.

**Key Points:**

*• There is a fair correlation between the Goutallier stage of the supraspinatus and infraspinatus muscle assessed on MRI and CT and visual assessment of fatty infiltration achieved on US.*

*• Shear wave elastography is not a valid tool for the determination of the amount of fatty infiltration or muscle atrophy.*

*• Shear wave elastography should not be used as a cheaper and less burdensome alternative for diagnostics in rotator cuff pathologies.*

## Introduction

Rotator cuff tears (RCTs) are the most common cause of shoulder pain in the aging population and the primary reason for shoulder surgery. When conservative therapy is unsuccessful, rotator cuff repair is a preferred surgical treatment for relieving complaints in people with a RCT [1, 2]. Muscle atrophy and fatty infiltration (FI) of the supraspinatus (SSP) and infraspinatus (ISP) muscle are factors predicting the post-surgical outcome and should therefore be assessed preoperatively. Moreover, the risk of tear recurrence is related to severe FI (Goutallier stage > 3) [3–6]. Currently, MRI is the gold standard in the preoperative assessment of the rotator cuff quality. The Goutallier and Warner grading systems are used to give an indication of the severity of FI and muscle atrophy of the rotator cuff, respectively. Several studies have shown promising results regarding the potential use of US and SWE as an alternative to this time-consuming, expensive, and burdensome method [7–12]. Using US and SWE for preoperative diagnostics in RCTs could be more time and cost-efficient. In previous studies, a converted MRI-based Goutallier classification was used for visual assessment of FI of the SSP and ISP on US. Using this scale, US findings correlated well with findings on MRI [8, 13, 14].

Additionally, SWE is a relatively new US technique capable of measuring velocities (in meters per second, m/s) which are supposed to reflect the composition of tissue. While the systematic review of Chiu et al [15] showed no significant differences in SWE values in tendons, there are encouraging results that SWE values can give an indication of the quality of the rotator cuff muscles.

A good to excellent intra- and inter-observer agreement (ICC = 0.7–0.970 and ICC = 0.45–0.948 respectively) was found for SWE in the SSP muscle [16–18]. A difference in SWVs between patients with or without tears is shown in several studies [16, 18, 19]. Thereby, Rosskopf et al [18] found a trend relating SWVs of the SSP muscle to the Goutallier stage on MRI. They also found a significant difference between the mean SWV and the presence of a positive tangent sign, indicating muscle atrophy of the SSP muscle.

For the US assessment of muscle atrophy, a reliable quantitative method was described by Khoury et al [20, 21]. The occupation ratio they calculated is thought to represent a reflection of the degree of muscle atrophy.

Based on these findings, the combination of these qualitative and quantitative methods for the assessment of muscle atrophy and FI using US and SWE may serve suitable alternative to MRI for preoperative assessment of the SSP and ISP muscle [14, 18–23].

US in combination with SWE could substantially reduce the burden and costs of the diagnostic process for patients with rotator cuff disorders. However, the validity of US and SWE compared to the current golden standard MRI for the assessment of muscle atrophy and FI has not yet been substantiated. To replace MRI for US and SWE in the diagnostic process, these techniques need to be both valid and reliable.

The aim of this study is to assess the validity of SWE in combination with US in the assessment of FI and muscle atrophy of the rotator cuff.

## Materials and methods

An institutional review board waiver was obtained for this validity study (METC-2020–2319).

### Study population

Between July 2020 and June 2021, patients older than 18 years, who had an available shoulder MRI or CT- scan within the last year were recruited in a single medical centre. Scans without the oblique sagittal plane or those performed with a contrast agent were excluded [8, 13, 18]. Eligible patients were invited for an US examination in the hospital.

### Imaging protocol MRI/CT

**Fig. 1:**
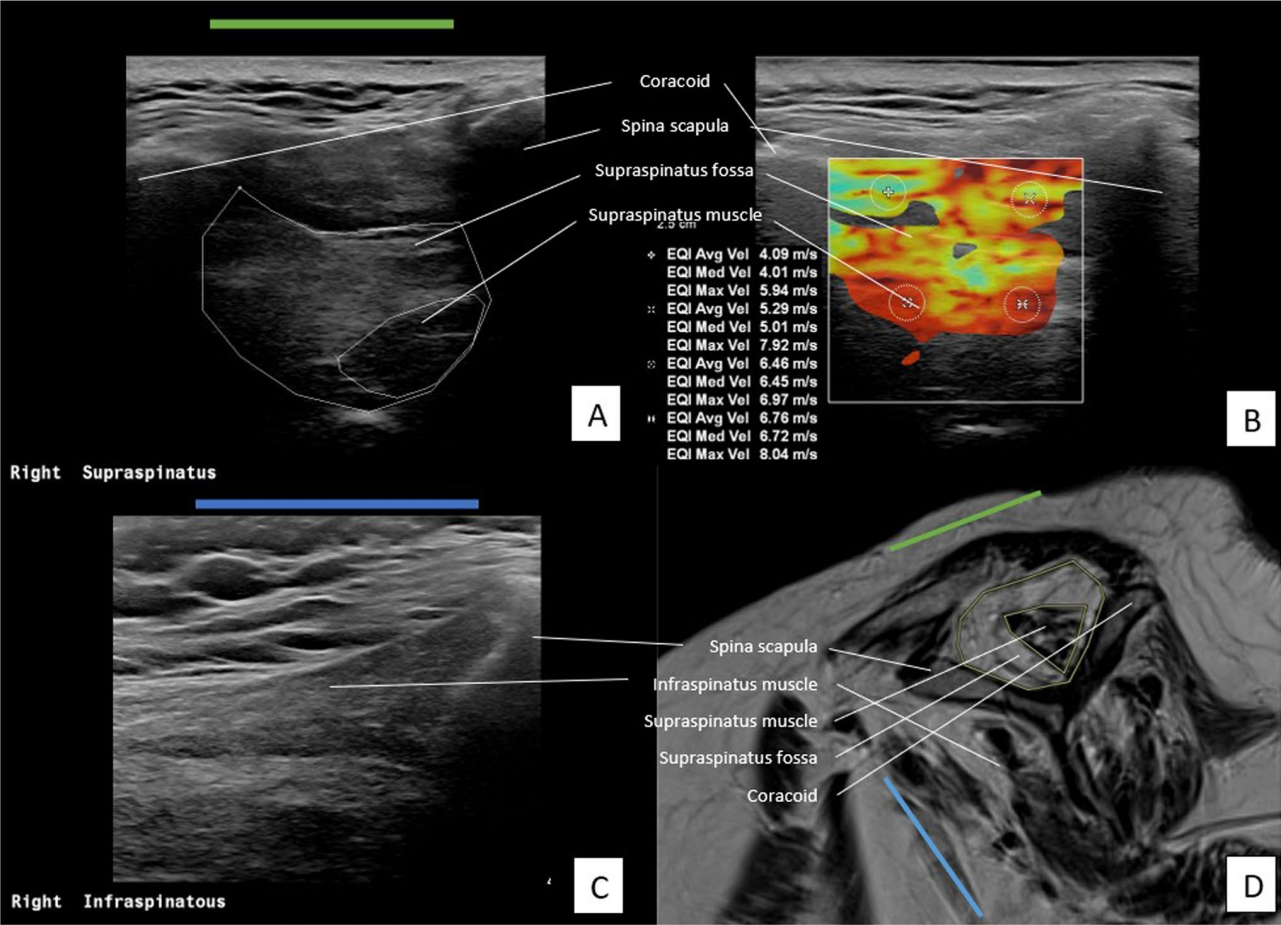
Assessment of SSP and ISP muscle on US and MRI of a patient with severe muscle atrophy and fatty infiltration of the SSP muscle. **A** US plane of SSP. **B **US plane of SSP with measurements of SWV in m/s in four quadrants. **C** US plane of ISP. **D** oblique-sagittal plane of MR image to assess SSP and ISP muscle

**Fig. 2:**
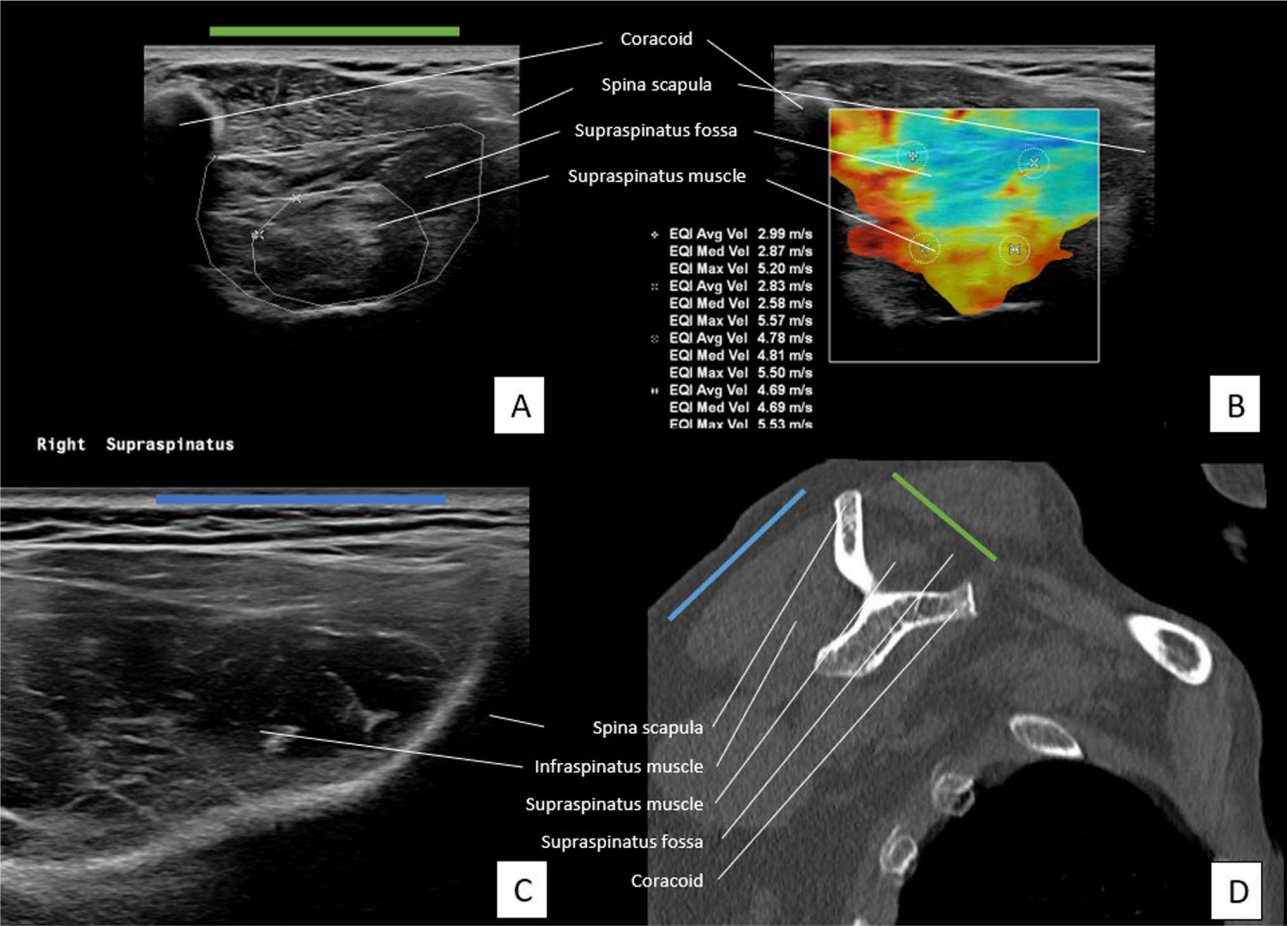
Assessment of SSP and ISP muscle on US and CT of patient severe muscle atrophy and fatty infiltration of the SSP muscle. **A** US plane of SSP. **B** US plane of SSP with measurements of SWV in m/s in four quadrants. **C** US plane of ISP. **D** oblique-sagittal plane of CT image to assess SSP and ISP muscle

MRI scans were performed using the Magnetom Aera 1.5 T or Sola 1.5 T, and CT scans were made using the Somatom Definition AS or Somatom go Top (Siemens Healthineers). The oblique-sagittal plane which crosses the scapula through the medial border of the coracoid process and offers a view of the SSP fossa, was used for evaluation of FI and atrophy (Fig. 1D). On MRI, a T2-weighted, Turbo Spin echo with the following parameters: TR = 5070 ms, TE = 72 ms, FOV 150 × 150, matrix 256 × 256, slice thickness 3 mm, 28 slices were used. CT exams were made in a supine position with a kV of 120, 200 quality ref. mAs, rotation time 1.0 s, slice thickness 2.0 mm, acquisition 128 × 0.6, and a pitch of 0.6. Reconstruction was made using a b60 sharp filter, 2.0 mm slice thickness, 2.0 mm increment, and a window width and window level of 2000/500. As shown in Fig. 2D, the oblique-sagittal plane is used for the assessment of CT scans. Patients were classified in the following manner based on the presence of a tear in the SSP, ISP, or subscapularis (SSC) muscle; ‘tear’, ‘no tear’, or ‘not assessable’ [24].

Both MRI and CT scans were used for the assessment of the Goutallier and Warner stages of the SSP and ISP muscle. The Goutallier classification, which consists of 5 stages, was used to quantify the amount of FI of the rotator cuff muscles. Grading was applied in the following manner: grade 0, no FI; grade 1, some fatty streaks; grade 2, less fat than muscle; grade 3, equal amounts of fat and muscle; grade 4, more fat than muscle [8, 25].

The method described by Thomazeau et al [20, 21] and Warner et al [26] was used for the assessment of muscle atrophy of the SSP muscle. For grading using the Warner classification, lines are drawn from the edge of the coracoid to the inferior scapular tip, from the inferior tip of the scapula to the spine, and from the scapular spine to the coracoid process on scan. If the muscle contour is convex outside both lines, there is no atrophy. If the muscle contour is even with the lines, there is mild atrophy. If the contour of the muscle is below the line, moderate atrophy is present. If there is barely any visible muscle, this is classified as severe atrophy [26].

Because of the lack of contrast between the SSP fossa and SSP muscle on CT scans only MR images were used for the quantitative assessment of muscle atrophy. The surfaces of the SSP fossa and muscle on the relevant plane were drawn and the values were entered in Castor Electronic Data Capture v.36.41 (Castor EDC, Ciwit BV) (Fig. 1D). The scans were reviewed by a musculoskeletal radiologist TK with 5 years of practical experience. The radiologist was blinded to the findings on the US.

### Imaging protocol US

The Philips EPIQ 7 with a linear transducer (eL18-4) was used to perform the US. The plane that was used in the assessment of MRI/CT scans was reproduced with the transducer. There where the SSP muscle is at the level of the most prominent muscle contour in an anteroposterior direction. A visualisation of the transverse plane (in relation to the long axis of the muscle) was achieved by turning the transducer perpendicular to the long axis of the muscle belly. In this plane the fossa–muscle ratio was calculated and SWVs were measured [18]. Images A and B in Figs. 1 and 2 show the US plane used to assess the SSP muscle. The position of the transducer in which the SSP muscle is assessed is shown as a green line.

Using the method by Rosskopf et al [18] the SWV was measured in four quadrants; anterosuperior (A), posterosuperior (B), anteroinferior (C), and posteroinferior (D). Per quadrant velocity in meters per second was measured (Fig. 1B and Fig. 2B). The mean SWV (mSWV) of all quadrants was used for statistical analyses. As Rosskopf et al [18] described, test–retest and interexaminer reliability of the mean of quadrants A and B were excellent. Because of the high reliability of these upper quadrants, the mean of quadrants A and B (AB) was measured and also used in comparison with scan results.

The SSP fossa- and muscle were measured by drawing the surface according to Khoury et al [20] (Fig. 1A and Fig. 2A)*.* The ratio between the cross-sectional area of the SSP muscle belly and that of its fossa was calculated and referred to as the occupation ratio. Visual assessment for grading by Goutallier staging was also applied to the US examination. Therefore, the Goutallier classification was collapsed to a three-point scale. The radiologist performed a visual assessment of the architecture and echogenicity for both the SSP and ISP muscle indicated by ‘Goutallier 0’, ‘Goutallier 1–2’, ‘Goutallier 3–4’, or ‘not assessable’ [13, 14].

Assessment of the ISP muscle (short axis) was obtained by turning the transducer perpendicular to the long axis of the muscle belly, with the scapular spine at the edge of the transducer. The blue line in images C and D in Figs. 1 and 2 indicates the position of the transducer to assess the ISP muscle. Blinded to the interpretation of the scan, US examination was performed by another musculoskeletal radiologist DI, with 9-year experience.

### Statistical analyses

Data was collected using Castor v.36.41. Data analysis was performed using IBM SPSS Statistic Software (version 26). Descriptive statistics were used to report the frequency of each recorded variable. The normality of the data was tested using the Shapiro–Wilk test and Q–Q plots. Correlation between US and scan results was tested using either a Pearson correlation coefficient or the Spearman Rank test, depending on the normality of the data. The following classification was applied to interpret the Spearman’s correlation coefficient (*r)*; poor (*r* < 0.29), fair (*r* = 0.30–0.59), moderate (*r* = 0.60–0.79), very strong (*r* = 0.80–0.99) or perfect (*r* = 1) (positive and negative rank correlations) [27]. Significant differences in mSWV or AB between the groups ‘tear’ or ‘no tear’ were calculated using the Mann–Whitney U Test.

## Results

Seventy-four patients were included in our study. In Table 1, descriptive results are shown. Seventy-eight shoulders were used for data analysis. The study population consisted of 38 males (51%) and 36 females (49%), the mean age of the participants was 63 ± 12. The mean body mass index (BMI) was 28 ± 4. In total, fifty-three MRI scans (68%) and 25 CT scans (32%) were available.

**Table 1:** Baseline characteristics of the study population

Characteristic	Patients (*n* = 74)
Age, mean ± SD (years)	63 ± 12
Gender (*n*) %	
Male	38 (51%)
Female	36 (49%)
BMI, mean ± SD (kg/m^2^)	28 ± 4
Imaging (*n*) %	
MRI	53 (68%)
CT	25 (32%)

All values are presented as numbers and valid percent unless indicated otherwise. SD = standard deviation; *BMI* body mass index

Based on the scan findings, forty-five shoulders (57,7%) were diagnosed with a tear in the SSP and 8 tears in the ISP tendon were found. In 26 cases (33%) the SSP tendon was not assessable for diagnosing a tear or no tear.

The occupation ratio was measured on all MRI scans. Median ratios per Goutallier grade and Warner stage are displayed in Tables 2 and 3.. A fair correlation between the ratio on US and MRI was found (*r* = 0.589). The ratio on US was fairly correlated with visual assessment of the Goutallier grade of the SSP muscle (*r* =  *− *0.550) and assessment of the Warner stage of the SSP muscle on MRI/CT (*r* =  − 0.516).

**Table 2:** Median values of shear wave velocities and occupation ratio per Goutallier grade of the SSP muscle

Goutallier stage		mSWV (in m/s)	AB (in m/s)	Occupation ratio US images	Occupation ratio MR images
Goutallier 0	*Median (n)*	4.6 (28)	4.1 (28)	0.67 (30)	0.58 (18)
	*SD*	0.53	0.59	0.21	0.33
	*Min–max*	3.23–5.22	2.29–4.86	0.19–1	0.38–1.93
Goutallier I	*Median (n)*	4.28 (16)	3.75 (16)	0.63 (17)	0.42 (13)
	*SD*	0.63	0.56	0.16	0.69
	*Min–max*	3.13–5.71	2.93–5.29	0.4–0.86	0.22–2.38
Goutallier II	*Median (n)*	4.26 (8)	4.18 (9)	0.45 (9)	0.36 (6)
	*SD*	0.56	0.48	0.15	0.083
	*Min–max*	3.73–5.39	3.37–4.79	0.17–0.61	0.21–0.42
Goutallier III	*Median (n)*	4.66 (10)	3.66 (10)	0.52 (10)	0.33 (7)
	*SD*	0.46	0.57	0.15	0.079
	*Min–max*	3.68–5.22	3.05–4.68	0.25–0.67	0.21–0.42
Goutallier IV	*Median (n)*	4.63 (10)	4.03 (10)	0.39 (12)	0.18 (9)
	*SD*	0.56	0.58	0.1	0.076
	*Min–max*	3.82–5.65	2.91–4.69	0.2–0.55	0.09–0.33

*SD* standard deviation; *mSWV* mean velocity of measurement A, B, C, and D (total surface of the fossa SSP); AB = mean velocity of A and B (upper two quadrants of the fossa SSP)

**Table 3:** Median values of shear wave velocities and occupation ratio per Warner stage of the SSP muscle

Warner stage		mSWV (in m/s)	AB (in m/s)	Occupation ratio US images	Occupation ratio MR images
None	*Median (n)*	4.45 (39)	4.04 (39)	0.67 (41)	0.55 (26)
	*SD*	0.59	6.04	0.21	0.52
	*Min–max*	3.13–5.71	2.29–5.29	0.19–1	0.29–2.38
Mild	*Median (n)*	4.71 (17)	3.92 (18)	0.45 (19)	0.33 (14)
	*SD*	0.51	0.53	0.17	0.07
	*Min–max*	3.73–5.65	3.05–4.79	0.17–0.83	0.21–0.42
Moderate	*Median (n)*	4.53 (12)	3.88 (12)	0.49 (13)	0.23 (9)
	*SD*	0.52	0.59	0.13	0.07
	*Min–max*	3.68–5.22	2.91–4.68	0.25–0.62	0.17–0.35
Severe	*Median (n)*	4.63 (4)	4.03 (4)	0.36 (5)	0.17 (4)
	*SD*	0.25	0.15	0.076	0.04
	*Min–max*	4.15–4.69	3.88–4.23	0.27–0.46	0.09–0.18

*SD* standard deviation; *mSWV* mean velocity of measurement A, B, C, and D (total surface of the fossa SSP); *AB* = mean velocity of A and B (upper two quadrants of the fossa SSP)

In Tables 2 and 3., median mSWV is reported per Goutallier and Warner stage. A poor correlation was found between mSWV on US and Goutallier grade of the SSP muscle on MRI/CT (*r* = 0.024) or Warner stage of the SSP muscle on MRI/CT (*r* = 0.07). Furthermore, the correlation between mSWV and ratio on MRI scan was poor (*r* =  − 0.116).

On US, the mean SWV of quadrants A and B (AB) was measured per patient. In Tables 2 and 3., median AB is reported per Goutallier and Warner stage. Poor correlation was found between AB and Goutallier grade (*r* =  − 0.103) or assessment of Warner stage on MRI/CT (*r* =  − 0.009). AB correlated poor with a ratio on MRI (*r* = 0.010) as well.

After the exclusion of obese patients (BMI > 30), a poor correlation between SWVs and scan results was found (*r* =  − 0.169 to 0.097).

The mean mSWV and mean AB in patients with RCTs are 4.44 m/s and 3.98 m/s respectively. For patients without tears, mean values are 4.87 m/s and 4.06 m/s. There is no difference found in mSWV and AB between ‘tear’ or ‘no tear’ group (resp. *p* = 0.057 and *p* = 0.716).

Correlations of visual assessment of Goutallier grade of the SSP and ISP muscle between US and MRI/CT were fair (resp. *r* = 0.582 and *r* = 0.574).

### Secondary outcomes

All scan results were measured on either MRI or CT images, depending on availability, except for the occupation ratio, which can only be measured reliably on MRI. After excluding CT data, a poor correlation was found between SWVs and Goutallier or Warner stage on MRI (*r* =  − 0.083 to 0.187). A fair correlation was found between the ratio on US and Goutallier or Warner stage on MRI (resp. *r* =  − 0.576 and -0.562). Correlations between visual assessment of FI using US and Goutallier grade of the SSP and ISP muscle on MRI were fair (resp. *r* = 0.557 and *r* = 0.651).

Goutallier stage of SSP muscle on MRI and ratio measured on MRI correlated moderate (*r* = 0.793). A very strong correlation (*r* = 0.858*)* was found between Warner stage on MRI and occupation ratio on MRI.

A poor correlation (*r* =  − 0.104–0.034) was found between visual assessment of FI of the SSP muscle on US and SWV values; mSWV and AB.

Including all available scans (MRI and CT), a very strong correlation (*r* = 0.880) was found between Goutallier and Warner stages.

## Discussion

The aim of this study was to investigate the validity of US in combination with SWE in the preoperative assessment of the rotator cuff. This is the first study that used the original Warner classification for grading muscle atrophy on scan to compare with SWVs and occupation ratio on US.

A poor correlation was found between SWVs and scan results for the assessment of FI and muscle atrophy. The occupation ratio on US showed a fair correlation with Warner and Goutallier’s stage on scan. Visual assessment of FI on US correlated fair with Goutallier stage on scan, both for the SSP and ISP muscle.

While our study focused on including a larger population of patients with a higher amount of FI or muscle atrophy, the lack of correlation is in line with a previous study by Rosskopf et al [18]. Although they did find a trend of a decreasing SWV at an increasing Goutallier stage on MRI and a negative correlation between SWV and the presence of a tangent sign, we found no evidence for these findings. A small population of patients with higher grades of FI (Goutallier 3–4) and patients with a positive tangent sign (both *n* = 10) in their study may have affected these results.

The difference in SWVs in patients with RCTs shown in other studies [16, 18] was not proven in our study. Lower mean mSWV and AB were found in the ‘tear’ group compared to the ‘no tear’ group but due to the small population (tear group; *n* = 7) we could not confirm this difference.

We found a fair correlation between a quantitative method for indicating muscle atrophy on US compared to MRI findings. These findings are not corresponding with the results of Khoury et al [20] which reported a good correlation between the occupation ratio on US and MRI (*r* = 0.98). A possible cause for this discrepancy in results could be a lower contrast between the surfaces of the SSP muscle and fossa in our study. Recently improved quality of transducers and the ability to smoothen images during processing could be negatively affecting the contrast. Moreover, our study includes more patients with a higher amount of FI (*n* = 25; *n* = 48). Defining the outline of the muscle could be more challenging due to a higher amount of FI. In addition, we specified the mean ratio on MR images for each patient group based on Warner or Goutallier stage. The mean ratio on MRI was higher in the case of a higher Goutallier or Warner stage. The very strong correlation (*r* = 0.858) between the occupation ratio on MRI and Warner stage suggests that ratio determination is a valid quantitative method for assessing muscle atrophy on MRI.

In this study, visual assessment of FI on US was achieved by evaluating architecture and echogenicity on real-time images of the muscle. Furthermore, the original stages of the Goutallier or Warner classification were used for the assessment of the scan. It should be noted that due to the use of this approach, our study was more specific than prior work which used converted scales and static images [13, 14]. A fair correlation found between visual assessment of the SSP and ISP muscle on US and scan results was not in line with obtained results in these studies. While it is suggested that CT images may underestimate the degree of FI [28], we could not find any clinical relevant differences in results after excluding CT images. An improved agreement was shown while using a dichotomous scale instead of a converted scale (3-point scale). Although the original 5 stages of Goutallier were used for an accurate comparison with SWE, this is not relevant for clinical practice. The clinically relevant distinction is made between patients with Goutallier stage 0–2 and those with Goutallier 3–4.

Based on these arguments and findings, US may be used to give an indication of the degree of FI.

Some limitations in our study are worth noting and could direct future research. We observed unexpected differences between US and scan findings. Two different musculoskeletal radiologists performed US and scan assessments, which could have introduced a bias.

We included patients who had an available shoulder MRI or CT scan within the last year, meaning a maximum time difference between scan and US of 12 months.

Increased FI is seen in patients with an RCT only after one year [6, 29, 30]. Therefore, it is unlikely that a clinically relevant difference in FI occurred between the time of scan and the time of US assessment in this study.

We obtained inconsistent measurements of SWVs, especially in the lower quadrants. The diffuse distribution of FI and the deeper anatomical location of the SSP may be a reasonable cause for this [18]. Due to poor validity and limitations of SWE in our study, we cannot prove its capability to assess FI or muscle atrophy in the SSP muscle.

In conclusion, SWE is not a valid method to measure the amount of FI or muscle atrophy of the SSP muscle. Visual assessment of FI according to Goutallier on US can give an indication of the degree of FI of the SSP or ISP muscle. Based on these findings, we can conclude that a combination of SWE and US should not replace MRI in the standard preoperative assessment of patients with RCTs.

## Abbreviations

AB: Mean shear wave velocity of upper quadrants A and B
BMI: Body mass index
FI: Fatty infiltration
ISP: Infraspinatus
mSWV: Mean shear wave velocity
RCT: Rotator cuff tear
SSC: Subscapularis
SSP: Supraspinatus
SWE: Shear wave elastography
SWV: Shear wave velocity

## Declarations

### Guarantor

The scientific guarantor of this publication is F.O. Lambers Heerspink.

### Statistics and biometry

No complex statistical methods were necessary for this paper.

### Methodology

• prospective

• diagnostic

• performed at one institution
